# Correlation of slow‐wave sleep with motor and nonmotor progression in Parkinson's disease

**DOI:** 10.1002/acn3.51975

**Published:** 2023-12-14

**Authors:** Jing Chen, Danhua Zhao, Baoyu Chen, Qi Wang, Yuan Li, Junyi Chen, Chaobo Bai, Xintong Guo, Xiaotong Feng, Xiaoyu He, Lin Zhang, Junliang Yuan

**Affiliations:** ^1^ Department of Neurology Peking University Sixth Hospital, Peking University Institute of Mental Health, NHC Key Laboratory of Mental Health (Peking University), National Clinical Research Center for Mental Disorders (Peking University Sixth Hospital), Peking University Beijing 100191 China; ^2^ PF Center of Excellence, Department of Neurology UC Davis Medical Center, UC Davis School of Medicine Sacramento California USA

## Abstract

**Objective:**

This study aimed to explore the association between slow‐wave sleep and the progression of motor and nonmotor symptoms in patients with PD.

**Methods:**

Data were collected from the Parkinson's Progression Markers Initiative study. Slow‐wave sleep, also known as deep non‐rapid eye movement (DNREM) sleep, was objectively assessed using the Verily Study Watch. Motor function was assessed using the Movement Disorder Society‐Unified Parkinson's Disease Rating Scale Part III score, Hoehn and Yahr stage, freezing of gait, motor fluctuations, and dyskinesia severity. Comprehensive assessments were conducted on nonmotor symptoms, including depression, anxiety, global cognitive function, and autonomic dysfunction. Statistical analyses involved repeated‐measures analysis of variance and linear regression.

**Results:**

A total of 102 patients with PD were included in the study, with a median follow‐up duration of 3.4 years. In the long DNREM sleep duration group (*n* = 55), better motor function (DNREM × time interaction: *F*_*(1,100)*_ = 4.866, *p* = 0.030), less severe sexual dysfunction (*p* = 0.026), and improved activities of daily living (*p* = 0.033) were observed at the last follow‐up visit compared with the short DNREM sleep duration group (*n* = 47). Reduced DNREM sleep duration is a risk factor for motor progression (*β* = −0.251, *p* = 0.021; 95% confidence interval = −0.465 to −0.038).

**Interpretation:**

The findings suggest an association between longer DNREM sleep duration and slower motor and nonmotor progression in patients with PD.

## Introduction

Sleep disturbances ranks among the most prevalent nonmotor symptoms of Parkinson's disease (PD), severely affecting patient quality of life and function.^1^, ^2^, ^3^ These disruptions often manifest in the prodromal stages and escalate with disease advancement.^4^ Recent discussions have highlighted the bidirectional link between PD and sleep disorders.^5^ Contrary to the traditional belief that sleep disturbances result from PD, emerging evidence proposes that they may also act as risk factors contributing to the onset and progression of PD.^6^, ^7^, ^8^, ^9^, ^10^

Mounting evidence suggests that slow‐wave sleep (SWS), also known as deep non‐rapid eye movement (DNREM) sleep, could play a pivotal role in neurodegenerative diseases.^11^ In mouse models of PD, sleep‐modulating treatments that enhanced slow waves exhibited a reduction in pathological alpha‐synuclein accumulation compared with the control groups, whereas non‐pharmacological sleep deprivation notably increased these pathological burdens.^8^ Studies employing polysomnography (PSG) assessing sleep in patients with PD found a correlation between deeper sleep and slower motor progression in PD. Additionally, reduced SWS was linked to cognitive decline in PD.^12^, ^13^ However, these studies were limited by sample size and lacked comprehensive data on nonmotor symptoms. They also quantified SWS based on a single night of PSG without prior habituation. However, further studies are required to confirm these results.

This study aimed to analyze whether DNREM sleep duration, objectively measured using the Verily Study Watch (VSW), correlates with motor and nonmotor progression in patients with PD from the Parkinson's Progression Markers Initiative (PPMI) cohort study.^14^, ^15^

## Methods

### Study design and participants

This study aimed to investigate the sleep patterns of patients with PD and the correlation between SWS and both motor and nonmotor progression using data from the PPMI study. The PPMI is an international, multicenter, longitudinal, observational study aimed at identifying biomarkers of PD progression in patients with de novo PD (diagnosed within 2 years). Eligibility criteria details for PPMI are accessible on their PPMI website (www.ppmi‐info.org)^15^. In the PPMI study, prodromal PD, unaffected by a known PD risk factor, is characterized by specific conditions such as LRRK2 mutations, GBA mutations, SNCA mutations, SNCA duplications, hyposmia, and rapid eye movement (REM) sleep behavior disorder. All participants in the PPMI provided written informed consent, and the study was approved by the Institutional Review Board of each study site.

Data retrieval from the PPMI website was performed on 13 March 2023. Sleep patterns were objectively evaluated using the VSW. First, participants who wore the VSW, comprising healthy controls (HCs), those with prodromal PD (pPD), and patients with PD, were enrolled to assess the clinical characteristics of sleep. Second, we explored the relationship between SWS and motor as well as nonmotor progression in patients with PD. The clinical visit just before the VSW usage was considered as the baseline for this investigation. We collected data on motor and nonmotor symptoms at both baseline and the final follow‐up visit. Patients with PD meeting the following criteria were included: (1) wearing the VSW for at least three consecutive nights for sleep assessment, and (2) availability of the Movement Disorder Society‐Unified Parkinson's Disease Rating Scale Part III (MDS‐UPDRS III) scores at baseline and the last follow‐up visit. The exclusion criterion encompassed undergoing deep brain stimulation (DBS) surgery during the follow‐up.

### Assessment of demographic and clinical characteristics

We assessed various demographic and clinical attributes, including age at baseline, sex, years of education, disease duration at baseline, and levodopa equivalent dose (LED) at baseline and the last follow‐up visit. Disease duration was defined as the time from symptom onset to baseline of this study. LED was calculated using the conversion formulae suggested by the MDS in 2023.^16^ Furthermore, patient activities of daily living were evaluated using the modified Schwab and England Activities of Daily Living (SEADL).^17^

### Assessment of sleep

DNREM sleep time and total sleep time were objectively quantified using the VSW, and the percentage of DNREM sleep time to total sleep time was calculated.^14^ The VSW, developed and validated by Verily data science team at Alphabet company, was provided to >800 US participants in the PPMI. The VSW is a wrist‐worn watch that effortlessly collects data on movement and various physiological and environmental measures throughout the day. Participants in the PPMI study who consented to wear the watch were included in the sub‐study, and there were no specific requirements regarding the duration of wearing the watch. Sleep comprises two fundamental stages: NREM sleep, which includes N1, N2, and N3, and REM sleep. Each 30‐second interval was categorized into one of five classes: wake, N1, N2, N3, and REM. The VSW algorithm merges the N1 and N2 classes into a single category termed light sleep, whereas N3 is identified as deep sleep. The VSW provides raw sensor data on sleep, including total sleep time, wake after sleep onset, sleep efficiency, number of awakenings, total NREM sleep time, total REM sleep time, DNREM sleep time, and light NREM (LNREM) sleep time. The sleep metrics algorithm involves a multistep process that utilizes accelerometer and photoplethysmography (PPG) signals from the VSW. It detects in‐bed/out‐of‐bed, sleep onset/offset, and the four sleep stages: wake, light sleep, deep sleep, and REM sleep. Sridhar et al. utilized two large public datasets, namely the Sleep Heart Health Study (SHHS) and the Multi‐Ethnic Study of Atherosclerosis Study (MESA), for the training, validation, and testing of the VSW algorithm. Additionally, an independent dataset consisting of 993 nights (993 subjects) from the Physionet Computing in Cardiology (CinC) dataset was used as the test set. The overall accuracy (Kappa κ) achieved was 78% (0.67), 80% (0.69), and 72% (0.55) for SHHS, MESA, and CinC, respectively. The algorithm demonstrated per‐class performance in terms of recall and precision as follows: wake‐(0.80, 0.86), LNREM‐(0.82, 0.74), DNREM‐(0.49, 0.68), REM‐(0.81, 0.76) on SHHS, and wake‐(0.74, 0.61), LNREM‐(0.76, 0.79), DNREM‐(0.48, 0.67), REM‐(0.76, 0.66) on CinC.^14^

Furthermore, the Epworth Sleepiness Scale, Rapid Eye Movement Sleep Behavior Disorder Screening Questionnaire, and scores of items 1.7 and 1.8 of the MDS‐UPDRS Part I were used to subjectively assess sleep.

### Assessment of motor symptoms

We assessed motor progression by examining changes in the MDS‐UPDRS III scores from baseline to the last follow‐up visit (median follow‐up time = 3.4 years). The severity of motor symptoms was determined using the Hoehn and Yahr stages. Motor phenotype classification (tremor dominant, postural instability/gait difficulty, or indeterminate) relied on established methods using the baseline MDS‐UPDRS III score.^18^ Freezing of gait (FOG), dyskinesia, and motor fluctuation were binary variables, based on the MDS‐UPDRS items 3.11, 4.1, or 4.3 (0 vs. ≥1). All patients were assessed in the “on” state.

### Assessment of nonmotor symptoms

Nonmotor experiences of daily living were evaluated using the total scores of MDS‐UPDRS Part I. Mood complaints were assessed using the 15‐item short form of the Geriatric Depression Scale (0–15, higher = worse) and the State–Trait Anxiety Inventory (20–80, higher = worse).^19^, ^20^ Global cognition was assessed with the Montreal Cognitive Assessment (0–30, higher = better).^21^ Dysautonomia was evaluated using the scores of the Scale for Outcomes in Parkinson's Disease for Autonomic Symptoms (SCOPA‐AUT).^22^ The SCOPA‐AUT consists of 25 items assessing various dysfunctions in gastrointestinal (7), urinary (6), cardiovascular (3), thermoregulatory (4), pupillomotor (1), and sexual (two items for men, two items for women) domains, with higher scores indicating worse symptoms.^22^

### Statistical analysis

We analyzed data collected at baseline and the last follow‐up visit. We compared groups using chi‐squared tests for categorical data, Student's *t*‐tests, or one‐way analysis of variance (with Bonferroni post hoc test) for normally distributed variables, Mann–Whitney *U* tests or Kruskal–Wallis test (with Bonferroni correction used for post hoc test) for nonparametric data. We divided the included patients with PD into two groups based on the median level of DNREM sleep time at baseline: those with long DNREM sleep duration (LDNREM) and those with short DNREM sleep duration (SDNREM). Subsequently, we compared the progression of motor and nonmotor symptoms between these groups. Repeated‐measures analysis of variance was employed to assess whether motor progression (assessed by change in MDS‐UPDRS III score during follow‐up) differed between the groups. Baseline and last follow‐up visit measures were used as within‐subject factors, and dichotomized DNREM sleep time was the between‐subject factor. We performed univariate and multivariate linear analyses, using backward linear regression analysis, to assess changes in the MDS‐UPDRS III score during follow‐up as dependent variables. Initially, we conducted a univariate linear regression involving factors such as age at baseline, sex, disease duration at baseline, follow‐up time, motor phenotype, LED at baseline, and DNREM sleep time at baseline. These factors were selected based on previously established associations and clinical insights. Subsequently, variables with *p* < 0.2 in the univariate models were included in the multivariate model. MDS‐UPDRS III scores at baseline were not part of the multivariate linear regression analyses to predict changes in the MDS‐UPDRS III score. We ensured that the residuals met all linear regression assumptions. Furthermore, we replaced the measurement of DNREM sleep time with the calculation of DNREM/total sleep time (%) and performed multivariate linear regression analyses. Statistical analyses were carried out using SPSS version 26 and GraphPad Prism version 9.0, considering *p* < 0.05 as significant.

## Results

### Comparison of sleep characteristics in HCs, participants with pPD, and patients with PD

**Figure 1:**
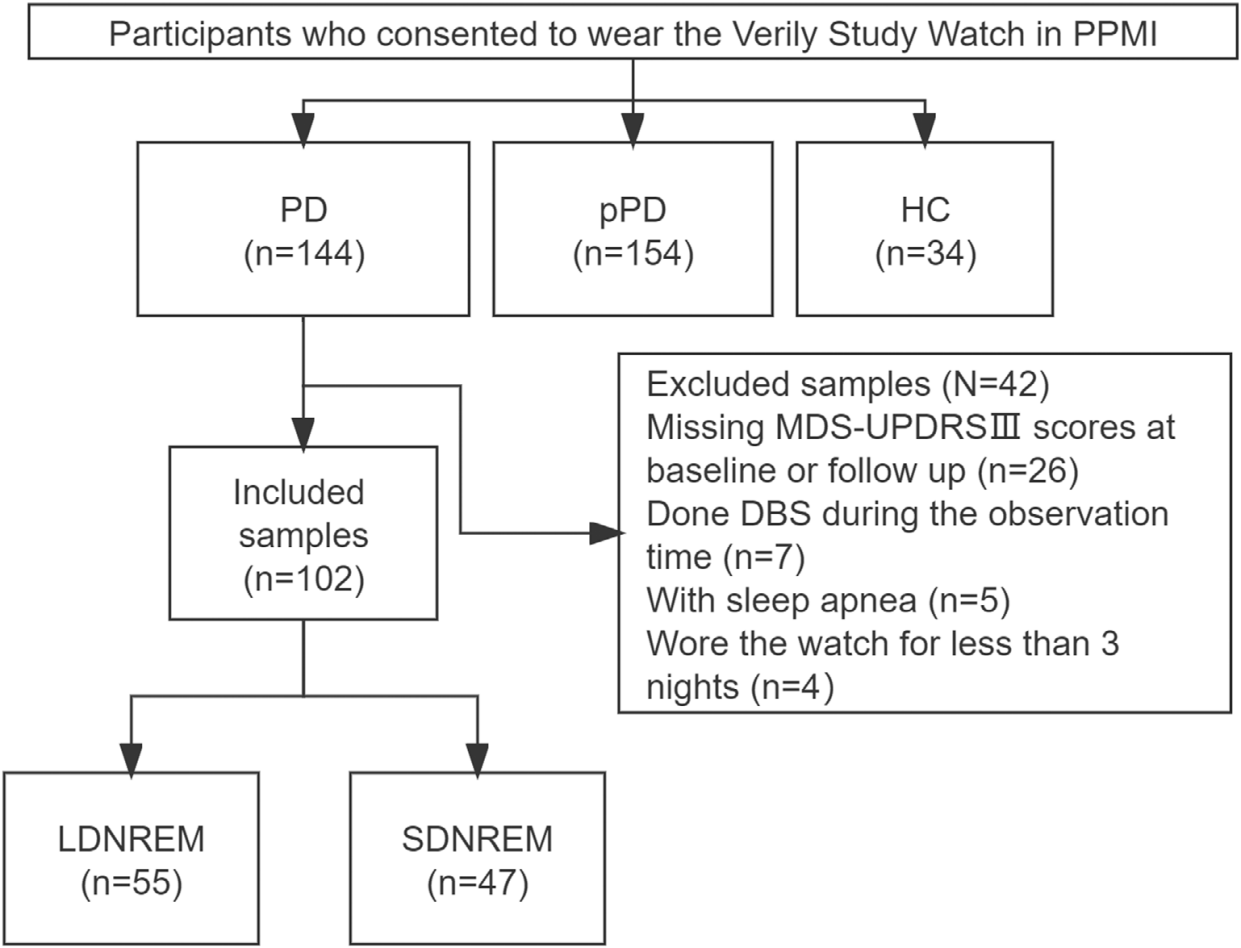
Flowchart of patient selection. HC, healthy control; LDNREM, long deep non‐rapid‐eye‐movement sleep time; PD, Parkinson's disease; pPD, prodromal Parkinson's disease; SDNREM, short deep non‐rapid eye movement sleep time.

We included 34 HCs, 154 participants with pPD, and 144 patients with PD from the PPMI database (Fig. 1). The baseline characteristics of each group are presented in Table 1. Compared with pPD participants and HCs, patients with PD exhibited shorter total sleep time, REM sleep time, DNREM sleep time, NREM/total sleep time (%), more awakenings, and longer wake time after sleep onset (*p* < 0.05, Table 1). No significant difference was found between pPD participants and HCs (*p* > 0.05, Table 1).

**Table 1:** Demographic and clinical characteristics of healthy controls, prodromal PD, and PD groups.

Characteristics	HCs	pPD	PD	*p* value
Sample size, *n*	34	154	144	
Male, *n* (%)	20 (58.8)	58 (37.7)	87 (60.4)	*p* < 0.001*** (pPD < PD, pPD < HC)
Age, years	66.9 ± 12.3	64.9 ± 7.2	68.9 ± 8.1	*p* < 0.001*** (pPD < PD)
GDS‐15 score	4.9 ± 0.8	5.4 ± 1.4	5.7 ± 1.6	*p* = 0.040* (HC < PD)
STAI score	94.0 ± 7.2	95.2 ± 4.3	90.3 ± 9.2	*p* = 0.001** (PD < HC, PD < pPD)
ESS score	4.8 ± 3.1	4.5 ± 3.1	7.7 ± 4.4	*p* < 0.001*** (HC < PD, pPD < PD)
RBDSQ score	2.5 ± 2.6	3.2 ± 2.7	5.7 ± 3.5	*p* < 0.001*** (HC < PD, pPD < PD)
Nights of wearing the watch, *n*	216 (31, 428)	204 (66, 336)	156 (52, 294)	*p* = 0.528
Total sleep time, min	353.0 ± 41.8	356.7 ± 42.0	322.67 ± 47.6	*p* = 0.001** (PD < HC, PD < pPD)
Wake time after sleep onset, min	35.0 ± 12.1	33.9 ± 11.3	40.0 ± 10.3	*p* < 0.001*** (pPD < PD)
Sleep efficiency, %	0.74 ± 0.13	0.72 ± 0.14	0.59 ± 0.14	*p* < 0.001*** (PD < HC, PD < pPD)
Number of awakenings, *n*	3 (2, 4)	3 (2, 4)	3 (3, 4)	*p* < 0.001*** (HC < PD, pPD < PD)
Total NREM sleep time, min	276.4 ± 31.4	280.6 ± 31.7	265.9 ± 37.4	*p* < 0.001*** (PD < pPD)
Total REM sleep time, min	76.7 ± 20.6	76.1 ± 18.3	56.8 ± 15.5	*p* < 0.001*** (PD < HC，PD < pPD)
DNREM sleep time, min	29.0 ± 15.4	31.7 ± 18.8	17.7 ± 36.9	*p* < 0.001*** (PD < HC, PD < pPD)
DNREM/total sleep time (%)	8.1 ± 4.0	8.9 ± 5.2	5.4 ± 3.1	*p* < 0.001*** (PD < HC, PD < pPD)
Light NREM sleep time, min	247.3 ± 33.3	249.0 ± 35.5	248.1 ± 36.9	*p* = 0.846

Data are presented as n (%), mean ± SD (range), or median (IQR).

DNREM, deep non‐rapid eye movement; ESS, Epworth Sleepiness Scale; GDS‐15, 15‐item short form of Geriatric Depression Scale; HCs, healthy controls; IQR, interquartile range; NREM, non‐rapid eye movement; PD, Parkinson's disease; pPD, prodromal Parkinson's disease; RBDSQ, rapid eye movement sleep Behavior Disorders Screening Questionnaire; REM, rapid eye movement; SD, standard deviation; STAI, State–Trait Anxiety Inventory.

* *p* < 0.05.

** *p* < 0.01.

*** *p* < 0.001.

### Demographic and clinical characteristics of patients with PD

In the PD group, 26 patients lacked the MDS‐UPDRS III scores recorded at baseline or the last follow‐up visit, and 7 patients underwent DBS surgery during the follow‐up period. Additionally, 5 patients had sleep apnea, and 4 had worn VSW for <3 days. Therefore, these patients were excluded from the study. Ultimately, a total of 102 patients with PD were selected to assess the association between DNREM sleep duration and motor and nonmotor progression (Fig. 1). Of these 102 patients with PD, 55 were categorized into the LDNREM sleep duration group, and 47 were in the SDNREM sleep duration group (Fig. 1). A higher proportion of males was noted in the SDNREM group (*p* = 0.003; Table 2).

**Table 2:** Demographic and clinical characteristics of all patients with PD and subgroups of LDNREM and SDNREM.

Demographic and Clinical Characteristics	All PD patients	LDNREM	SDNREM	*p* value
Sample size, *n*	102	55	47	
Age at baseline, years	67.1 ± 8.2	66.7 ± 8.7	67.6 ± 7.6	0.571
Male, *n* (%)	65 (63.7)	27 (49.1)	38 (80.9)	0.003**
Education, years	17.5 ± 3.9	16.8 ± 2.8	18.4 ± 4.8	0.262
Disease duration at baseline, years	6.0 ± 1.8	5.8 ± 1.6	6.4 ± 2.0	0.200
Wearing a watch to baseline, m	12 (6, 12)	12 (6, 12)	12 (6, 12)	0.156
Follow‐up time, years	3.4 (2.8, 3.9)	3.6 (2.9, 4.0)	3.1 (2.1, 3.9)	0.059
Nights of wearing the watch, *n*	138 (49, 285)	207 (60, 409)	124 (39, 255)	0.063
Total sleep time, min	322.7 ± 43.3	329.2 ± 39.5	315.1 ± 46.5	0.113
Wake time after sleep onset, min	38.7 ± 10.1	37.2 ± 10.3	40.4 ± 9.7	0.054
Sleep efficiency, %	59.5 ± 13.3	63.3 ± 13.1	55.0 ± 12.2	0.002**
Number of awakenings, *n*	3 (3–4)	3 (3–4)	3 (3–4)	0.617
Total NREM sleep time, min	265.6 ± 33.6	268.5 ± 31.4	262.3 ± 36.1	0.489
Total REM sleep time, min	57.1 ± 14.8	60.8 ± 12.7	52.8 ± 16.1	0.005**
DNREM sleep time, min	17.4 ± 9.8	24.0 ± 8.2	9.7 ± 4.2	<0.001***
DNREM/total sleep time (%)	5.4 ± 3.1	7.4 ± 2.7	3.1 ± 1.3	<0.001***
Light NREM sleep time, min	248.2 ± 34.1	244.5 ± 32.6	252.6 ± 35.5	0.235
LED, mg/d				
Baseline (*n* = 102)	886.7 ± 575.2	829.2 ± 594.0	954.0 ± 551.1	0.148
Follow‐up (*n* = 45)	924.7 ± 474.7	857.8 ± 531.9	1001.1 ± 398.6	0.142
SEADL score				
Baseline (*n* = 102)	84.2 ± 8.1	84.6 ± 8.4	83.7 ± 7.8	0.701
Follow‐up (*n* = 102)	81.6 ± 13.3	83.8 ± 12.7	79.0 ± 13.7	0.033*

Data are presented as n (%), mean ± SD (range), or median (IQR).

DNREM, deep non‐rapid eye movement; IQR, interquartile range; LDNREM, long deep non‐rapid eye movement sleep time; LED, levodopa equivalent dose; NREM, non‐rapid eye movement; PD, Parkinson's disease; REM, rapid eye movement; SD, standard deviation; SDNREM, short deep non‐rapid eye movement sleep time; SEADL, modified Schwab England Activities of Daily Living.

* *p* < 0.05.

** *p* < 0.01.

*** *p* < 0.001.

The median absolute time between the baseline in this study and the VSW was 12 months, with a median of 138 nights the watch was worn. The median follow‐up time was 3.4 years. Patients with PD and SDNREM had lower sleep efficiency and shorter total REM sleep time than those in the LDNREM group (*p* < 0.01; Table 2). Additionally, patients with PD in the SDNREM group exhibited lower SEADL scores than those in the LDNREM group at the last follow‐up visit (*p* = 0.033; Table 2), with no significant difference observed at baseline (*p* = 0.701; Table 2). No significant differences were observed in age, disease duration, years of education, follow‐up time, and LED at baseline or the last follow‐up visit between the two groups (*p* > 0.05; Table 2).

### Motor symptoms in SDNREM and LDNREM

Patients with PD and SDNREM showed higher MDS‐UPDRS III scores at the last follow‐up visit than those in the LDNREM group (*p* = 0.011; Table 3), although no difference observed at baseline (*p* = 0.205; Table 3). No significant difference was noted in motor phenotype at baseline (*p* = 0.684; Table 3). No significant difference was observed between the two groups in the Hoehn and Yahr stage, FOG, dyskinesia, or motor fluctuation at baseline and the last follow‐up visit (*p* > 0.05; Table 3).

**Table 3:** Motor symptoms of all patients with PD and subgroups of LDNREM and SDNREM.

Motor symptoms	All PD patients	LDNREM	SDNREM	*p* value
Sample size, *n*	102	55	47	
Motor phenotype, indeterminate/TD/PIGD, *n*	9/61/32	4/32/19	5/29/13	0.684
MDS‐UPDRS III score				
Baseline	22.5 ± 9.8	21.4 ± 9.3	23.8 ± 10.4	0.205
Follow‐up	27.5 ± 12.7	24.4 ± 10.6	31.2 ± 14.1	0.011*
Hoehn and Yahr stage				
Baseline (*n* = 102)	2 (2–2)	2 (2–2)	2 (2–2)	0.987
Follow‐up (*n* = 101)	2 (2–2)	2 (2–2)	2 (2–2)	0.261
FOG, *n* (%)				
Baseline	10 (9.8)	4 (7.3)	6 (12.8)	0.507
Follow‐up	27 (26.5)	15 (27.3)	12 (25.5)	0.843
Dyskinesia, *n* (%)				
Baseline	25 (24.8)	11 (20.4)	14 (29.8)	0.274
Follow‐up	70 (69.3)	35 (64.8)	35 (74.5)	0.294
Motor fluctuation, *n* (%)				
Baseline	63 (63.0)	31 (58.5)	32 (68.1)	0.321
Follow‐up	80 (81.6)	40 (78.4)	40 (85.1)	0.394

Data are presented as n (%), mean ± SD (range), or median (IQR).

FOG, freezing of gait; IQR, interquartile range; LDNREM, long deep non‐rapid eye movement sleep time; MDS‐UPDRS III, Movement Disorder Society‐sponsored revision of the Unified Parkinson's Disease Rating Scale Part III; PD, Parkinson's disease; PIGD, postural instability/gait difficulty; SD, standard deviation; SDNREM, short deep non‐rapid eye movement sleep time; TD, tremor dominant.

* *p* < 0.05.

### Nonmotor symptoms in SDNREM and LDNREM

Patients with PD and SDNREM had significantly higher scores on the SCOPA‐AUT sexual domain than patients with LDNREM at the last follow‐up visit (*p* = 0.026; Table 4), with no difference observed at baseline (*p* = 0.067; Table 4). No significant differences were found in the scores on the scales for depression, anxiety, global cognition, sleep, and other domains of the SCOPA‐AUT between the two groups (*p* > 0.05; Tables 4 and S1).

**Table 4:** Nonmotor symptoms of all patients with PD and subgroups of LDNREM and SDNREM.

Nonmotor symptoms	All PD patients	LDNREM	SDNREM	*p* value
MDS‐UPDRS I total score				
Baseline (*n* = 102)	10.5 ± 5.5	10.6 ± 5.8	10.4 ± 5.2	0.819
Follow‐up (*n* = 102)	12.0 ± 6.6	12.0 ± 6.5	11.9 ± 6.8	0.989
GDS‐15 score				
Baseline (*n* = 77)	5.7 ± 1.4	5.6 ± 1.5	5.8 ± 1.4	0.521
Follow‐up (*n* = 98)	6.2 ± 1.6	6.2 ± 1.8	6.2 ± 1.5	0.779
STAI score				
Baseline (*n* = 77)	91.7 ± 8.2	92. 8 ± 8.9	90.6 ± 7.3	0.350
Follow‐up (*n* = 98)	91.0 ± 8.6	91.8 ± 8.9	90.1 ± 8.2	0.367
MDS‐UPDRS I item 1.7				
Baseline (*n* = 101)	1.7 ± 1.2	1.9 ± 1.2	1.5 ± 1.2	0.123
Follow‐up (*n* = 101)	1.6 ± 1.2	1.7 ± 1.2	1.5 ± 1.2	0.295
MDS‐UPDRS I item 1.8				
Baseline (*n* = 101)	1.5 ± 0.9	1.5 ± 0.9	1.6 ± 1.0	0.675
Follow‐up (*n* = 101)	1.5 ± 1.0	1.5 ± 0.9	1.4 ± 1.1	0.433
ESS score				
Baseline (*n* = 77)	8.4 ± 5.0	8.0 ± 4.3	9.0 ± 5.6	0.606
Follow‐up (*n* = 98)	8.1 ± 4.2	8.3 ± 4.1	7.9 ± 4.3	0.582
RBDSQ score				
Baseline (*n* = 77)	5.6 ± 3.4	5.5 ± 3.4	5.8 ± 3.4	0.712
Follow‐up (*n* = 98)	5.8 ± 3.8	5.2 ± 3.4	6.4 ± 3.1	0.140
MoCA score				
Baseline (*n* = 72)	26.4 ± 3.5	26.5 ± 2.9	26.2 ± 4.1	0.678
Follow‐up (*n* = 64)	26.3 ± 3.9	26.8 ± 3.2	25.7 ± 4.2	0.323
SCOPA‐AUT total score				
Baseline (*n* = 77)	16.5 ± 8.4	15.6 ± 8.0	17.5 ± 8.7	0.163
Follow‐up (*n* = 54)	17.1 ± 9.5	15.3 ± 9.5	18.8 ± 9.4	0.158
SCOPA‐AUT sexual domain				
Baseline (*n* = 77)	1.5 ± 1.7	1.3 ± 1.6	1.8 ± 1.6	0.067
Follow‐up (*n* = 54)	2.1 ± 2.0	1.5 ± 1.9	2.7 ± 2.0	0.026*

Data are presented as mean ± SD (range).

ESS, Epworth Sleepiness Scale; GDS‐15, 15‐item short form of Geriatric Depression Scale; LDNREM, long deep non‐rapid eye movement sleep duration; MDS‐UPDRS I, Movement Disorder Society‐sponsored revision of the Unified Parkinson's Disease Rating Scale Part I; MoCA, montreal cognitive assessment; PD, Parkinson's disease; RBDSQ, Rapid eye movement sleep Behavior Disorders Screening Questionnaire; SCOPA‐AUT, The Scale for Outcomes in Parkinson's disease for Autonomic symptoms; SD, standard deviation; SDNREM, short deep non‐rapid eye movement sleep duration; STAI, State–Trait Anxiety Inventory.

* *p* < 0.05.

### Association of SWS with motor progression

**Figure 2:**
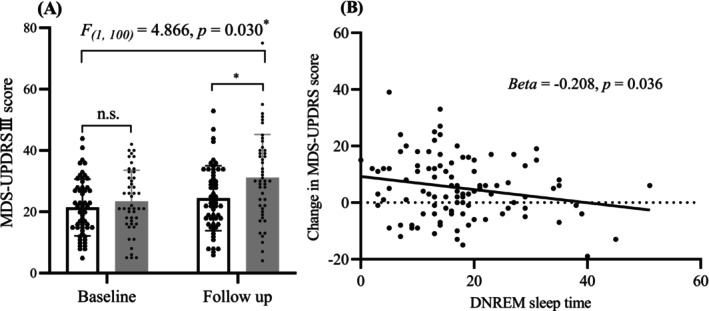
Longer deep non‐rapid eye movement (DNREM) sleep time was associated with slower motor progression in Parkinson's disease (PD). (A) Repeated‐measures analysis of variance revealed the progression of motor symptoms was slower in patients in the long DNREM group (marked white) compared with those in the short DNREM group (marked gray) (DNREM × time interaction: *F*_*(1,100)*_ = 4.866, *p* = 0.030). *p < 0.05. n.s. = not significant. (B) Baseline DNREM sleep duration is plotted against the change in MDS‐UPDRS III scores between baseline and the last follow‐up visit in 102 patients with PD. Longer DNREM sleep duration predicted a slower progression of motor symptoms (*β* = −0.208, *p* = 0.036; 95% confidence interval = −0.402 to −0.014, indicated by dark line).

Repeated‐measures analysis of variance showed that the progression of motor symptoms was slower in patients in the LDNREM group than those in the SDNREM group (DNREM × time interaction: *F*_*(1,100)*_ = 4.866, *p* = 0.030) (Fig. 2A).

In univariate linear analyses, longer DNREM sleep duration predicted slower motor progression in patients with PD (*β* = −0.208, *p* = 0.036; 95% confidence interval (*CI*) = −0.402 to −0.041) (Table 5, Fig. 2B). In multivariate linear analyses, reduced DNREM sleep duration is a risk factor for motor progression (*β* = −0.251, *p* = 0.021; 95% *CI* = −0.465 to −0.038) (Table 5). Furthermore, when DNREM sleep duration was replaced by the calculation of DNREM/total sleep time (%), similar results were obtained (*β* = −75.387, *p* = 0.031; 95% *CI* = −143.552 to −7.221, Table S2).

**Table 5:** Association between baseline clinical characteristics and changes in MDS‐UPDRS III scores during follow‐up in patients with PD.

Characteristics	Change of MDS‐UPDRS III score			
	Univariate analysis		Multivariate analysis	
	*β*	*p* value	*β*	*p* value
Age at baseline	0.168	0.207	NA	NA
Sex				
Male	−0.737	0.745	NA	NA
Female	Ref			
Disease duration at baseline	−0.507	0.452	NA	NA
Follow‐up time	−0.016	0.876	NA	NA
Motor phenotype				
Intermediate	2.634	0.505	NA	NA
PIGD	−0.189	0.938	NA	NA
TD	Ref			
LED at baseline	−0.004	0.047*	−0.004	0.027*
DNREM sleep time	−0.208	0.036*	−0.251	0.021*

DNREM, deep non‐rapid eye movement; LED, levodopa equivalent dose; MDS‐UPDRS III, the Movement Disorder Society‐sponsored revision of the Unified Parkinson's Disease Rating Scale Part III; PD, Parkinson's disease; PIGD, postural instability/gait difficulty; TD, tremor dominant.

* *p* < 0.05.

## Discussion

Our study revealed that a longer DNREM sleep duration was associated with slower motor and nonmotor progression in patients with PD, over a median follow‐up of 3.4 years. In the long DNREM sleep duration group, we observed improved motor function, lesser severity in sexual dysfunction, and enhanced activities of daily living during the last follow‐up visit compared with in the short DNREM sleep duration group. Reduced DNREM sleep duration emerged as a risk factor for motor progression.

The intimate connection between sleep and neurodegenerative diseases has garnered increased attention recently.^11^, ^23^, ^24^ The prevailing belief is that pathology of PD and various PD‐related motor or nonmotor symptoms trigger sleep disturbances in patients with PD. Recent research has demonstrated that poor sleep is related to a higher risk of PD and can exacerbate PD progression.^3^, ^7^, ^13^, ^25^, ^26^, ^27^, ^28^ However, in most of these studies, sleep assessments relied on subjective measures, lacking objective results. One PSG study indicated that deeper SWS might be related to a more favorable PD prognosis^13^. Our findings further support this framework, showing that extended SWS correlated with slower motor progression, reinforcing the proposed bidirectional relationship between sleep and PD.^5^ These effects could stem from the activation of GABAergic interneurons and the facilitation of glymphatic clearance during SWS.^29^, ^30^ The inhibition of neuronal activity might reduce alpha‐synuclein release, and glymphatic clearance could eliminate extracellular alpha‐synuclein, potentially slowing its propagation.^30^, ^31^, ^32^ Recent evidence has also highlighted that sleep deprivation leads to increased alpha‐synuclein and phosphorylated alpha‐synuclein in CSF.^32^, ^33^ Enhancing slow waves in sleep reduces pathological alpha‐synuclein accumulation in PD mouse models by boosting glymphatic clearance and regulating proteostatic processes.^8^

Research on the relationship between SWS and nonmotor symptoms in patients with PD is lacking. Our assessment encompassed depression, anxiety, global cognition, and autonomic function in both SDNREM and LDNREM groups. Similar to the findings in healthy individuals,^34^ patients with PD and SDNREM experienced severe sexual dysfunction during follow‐up. However, we detected no significant difference in global cognition between the two groups. This contrasts with previous studies,^12^, ^35^ and may be attributed to differences in study design, participants, and SWS assessment. Moreover, no severe depression or anxiety was observed in the SDNREM group.

Our study had several strengths. First, an objective evaluation of sleep was conducted using VSW across a median follow‐up period of 138 nights, offering a more comprehensive portrayal of sleep characteristics. Second, we extensively explored the correlation between SWS and motor and nonmotor symptoms in patients with PD. All data were derived from a substantial longitudinal cohort study (PPMI). To the best of our knowledge, this is the first SWS study to comprehensively evaluate motor and nonmotor PD symptoms simultaneously.

We acknowledge several limitations in this study. First, our research design was constrained by the available data from the PPMI. We observed sex differences between the LDNREM and SDNREM groups, consistent with previous research finding^13^, ^36^. The potential influence of sex on the differences in sexual dysfunction between these groups cannot be disregarded. Additionally, due to the limited availability of the MDS‐UPDRS III OFF score in PPMI, we used the MDS‐UPDRS III ON score to estimate PD motor progression, although the MDS‐UPDRS III OFF score might offer a more accurate assessment. The median time from baseline to VSW usage was 12 months, a considerable duration. As SWS is not anticipated to remain static, this study holds the potential for misclassifying LDNREM and SDNREM at the baseline. Further validation of our findings through large‐scale prospective studies is warranted. Second, the heterogeneity among patients with PD with varying disease durations might have affected our results, as it is possible that the precise association of SWS with progression could differ across different PD disease stages. Third, the VSW has not been validated in patients with PD. Sleep stage determination using heart rate by VSW might be influenced by factors such as the presence of atrial fibrillation and nocturnal motor function in patients with PD. Although three participants had atrial fibrillation in this study, we conducted univariate and multivariate linear analyses after excluding patients with PD with atrial fibrillation, and consistent results were obtained (data not shown). Despite these limitations, this work stands as one of the few studies examining the relationship between SWS and PD progression over a relatively long follow‐up period.

In conclusion, this study established that longer SWS correlated with slower motor progression, reduced severity of sexual dysfunction, and improved daily living activities. Our findings lend support to the potential value of SWS enhancement therapy in decelerating PD progression.

## Author Contributions

Conception and design: Jing Chen, Junliang Yuan, and Lin Zhang. Administrative support: Junliang Yuan and Lin Zhang. Collection and assembly of data: Jing Chen, Danhua Zhao, Baoyu Chen, Qi Wang, Yuan Li, Chaobo Bai, Junyi Chen, Xintong Guo, Xiaotong Feng, and Xiaoyu He. Data analysis and interpretation: Jing Chen. Manuscript writing: All authors. Final approval of manuscript: All authors. The final version has been revised by: Junliang Yuan, Lin Zhang, and Jing Chen.

## Funding information

This study was supported by the National Natural Science Foundation of China (82071552, 22376006) and the Chinese Academy of Sciences Grant (JCTD‐2021‐06).

## Conflicts of interest

The authors declare that the research was conducted in the absence of any commercial or financial relationships that could be construed as a potential conflict of interest.

## Supporting information


Table S1.
