# Supplementary material for: Correlation of slow‐wave sleep with motor and nonmotor progression in Parkinson's disease
Source: Ann Clin Transl Neurol. 2023 Dec 14;11(3):554–63. doi: 10.1002/acn3.51975 (PMC10963280; doi:10.1002/acn3.51975)
Supplement: Supplementary file 1 — Table S1. [file ACN3-11-554-s001.docx]

**Association between Slow-Wave Sleep and Motor and Nonmotor Progression in Parkinson Disease**

**Supplementary tables:**

**Table S1.** The SCOPA-AUT of all patients with PD and subgroups of LDNREM and SDNREM.

**Table S2.** Association between baseline clinical characteristics with change of MDS-UPDRS Ⅲ score during follow-up in patients with PD.

**Table S1.** The SCOPA-AUT of all patients with PD and subgroups of LDNREM and SDNREM.

| Non-motor symptoms | All PD patients | LDNREM | SDNREM | *p* value |
| --- | --- | --- | --- | --- |
| SCOPA-AUT | | | | |
| total score | | | | |
| Baseline (n = 77) | 16.5 ± 8.4 | 15.6 ± 8.0 | 17.5 ± 8.7 | 0.163 |
| Follow-up (n = 54) | 17.1 ± 9.5 | 15.3 ± 9.4 | 18.9 ± 9.4 | 0.317 |
| Gastrointestinal domain |  |  |  |  |
| Baseline (n = 77) | 3.7 ± 2.5 | 3.6 ± 2.5 | 3.9 ± 2.5 | 0.650 |
| Follow-up (n = 54) | 3.6 ± 2.3 | 3.2 ± 2.4 | 4.0 ± 2.3 | 0.191 |
| Urinary |  |  |  |  |
| Baseline (n = 77) | 5.8 ± 3.1 | 5.4 ± 2.9 | 6.2 ± 3.3 | 0.345 |
| Follow-up (n = 54) | 6.0 ± 3.6 | 5.5 ± 3.3 | 6.5 ± 3.9 | 0.369 |
| Cardiovascular |  |  |  |  |
| Baseline (n = 77) | 1.0 ± 1.5 | 1.1 ± 1.5 | 1.0 ± 1.6 | 0.406 |
| Follow-up (n = 54) | 1.1 ± 1.5 | 0.9 ± 1.3 | 1.2 ± 1.7 | 0.682 |
| Thermoregulatory |  |  |  |  |
| Baseline (n = 77) | 2.9 ± 2.7 | 2.9 ± 3.0 | 2.9 ± 2.4 | 0.778 |
| Follow-up (n = 54) | 2.7 ± 2.5 | 2.8 ± 2.8 | 2.5 ± 2.3 | 0.765 |
| Pupillomotor |  |  |  |  |
| Baseline (n = 77) | 0.7 ± 0.9 | 0.8 ± 0.9 | 0.7 ± 0.9 | 0.672 |
| Follow-up (n = 54) | 0.8 ± 0.9 | 0.8 ± 1.0 | 0.8 ± 0.9 | 0.522 |
| sexual domain | | | | |
| Baseline (n = 77) | 1.5 ± 1.7 | 1.3 ± 1.6 | 1.8 ± 1.6 | 0.067 |
| Follow-up (n = 54) | 2.1 ± 2.0 | 1.5 ± 1.9 | 2.7 ± 2.0 | 0.026^*^ |

Data are presented as mean ± SD (range).

SCOPA-AUT, the Scale for Outcomes in Parkinson’s disease for Autonomic symptoms; PD, Parkinson’s Disease; LDNREM, long deep Non-Rapid Eye Movement sleep duration; SDNREM, short deep Non-Rapid Eye Movement sleep duration; SD, standard deviation.

^*^ *p* < 0.05.

**Table S2.** Association between baseline clinical characteristics with the change of MDS-UPDRS Ⅲ score during follow-up in patients with PD.

| Characteristics | MDS-UPDRS Ⅲ score at follow-up | | | | | |
| --- | --- | --- | --- | --- | --- | --- |
|  | Univariate analysis | |  | | Multivariate analysis | |
|  | *β* | *p* value | |  | *β* | *p* value |
| Age at baseline | 0.168 | 0.207 | |  | NA | NA |
| Sex |  |  | |  |  |  |
| Male | -0.737 | 0.745 | |  | NA | NA |
| Female | Ref |  | |  |  |  |
| Disease duration at baseline | -0.507 | 0.452 | |  | NA | NA |
| Follow-up time | -0.016 | 0.876 | |  | NA | NA |
| Motor phenotype |  |  | |  |  |  |
| Intermediate | 2.634 | 0.505 | |  | NA | NA |
| PIGD | -0.189 | 0.938 | |  | NA | NA |
| TD | Ref |  | |  |  |  |
| LED at baseline | -0.004 | 0.047^*^ | |  | -0.004 | 0.039^*^ |
| DNREM/total sleep time (%) | -74.165 | 0.036^*^ | |  | -75.387 | 0.031^*^ |

PD, Parkinson’s Disease; MDS-UPDRS Ⅲ, the Movement Disorder Society-sponsored revision of the Unified Parkinson’s Disease Rating Scale Part Ⅲ; PIGD, postural instability/gait difficulty; TD = tremor dominant; LED, levodopa equivalent dose; DNREM, deep Non-Rapid Eye Movement sleep duration.

^*^ *p* < 0.05.
